# Constipation and Cigarette Smoking Are Independent Influences for Parkinson’s Disease

**DOI:** 10.7759/cureus.21689

**Published:** 2022-01-28

**Authors:** Steven Lehrer, Peter H Rheinstein

**Affiliations:** 1 Radiation Oncology, Icahn School of Medicine at Mount Sinai, New York, USA; 2 Family Medicine, Severn Health Solutions, Severna Park, USA

**Keywords:** parkinson's disease, diterpenes, risk factors, smoking, constipation

## Abstract

Background

Tobacco smokers have reduced Parkinson’s disease (PD) risk. Some patients with PD experience constipation long before they develop mobility problems, and constipation is a frequent complaint of people who try to stop smoking. Recently, the gut microbiome has been implicated in PD.

Methods

In the present study, we analyzed the relationship between smoking and constipation in subjects with PD and controls. We wished to determine whether the effects of smoking and constipation were independent or whether they might be interrelated. To evaluate the relationship, we used a cohort of subjects from the UK Biobank (UKB).

Results

In 501,174 subjects, the decreased risk of Parkinson’s disease with increased smoking was significant (p < 0.001, two-tailed Fisher’s exact test). The increased risk of constipation in subjects with PD was significant (p = 0.001, two-tailed Fisher’s exact test). Logistic regression was performed; sex, age, constipation, and smoking were the independent variables, and PD present or absent was the dependent variable. The PD odds ratio (OR) for males was 1.790 (95% confidence interval (CI): 1.629-1.966) times that for females, indicating that PD is more common in men. The risk of PD increased by 1.140 (95% CI: 1.131-1.149) with every year of age. Constipation increased the risk of PD by 4.043 (95% CI: 1.901-8.599). Smoking diminished PD risk by 0.772 (95% CI: 0.690-0.863). Drinking coffee was associated with a reduced risk of PD (OR: 0.815 (95% CI: 0.730-0.909). Drinking tea reduced PD risk by 0.979 (95% CI: 0.962-0.997) for each cup per day. The effects of sex, age, constipation, smoking, drinking coffee, and drinking tea were independent and significant.

Conclusion

Our analysis suggests that the favorable effect of smoking on PD is independent of the detrimental effect of constipation. Smoking reduces PD risk because it not only stimulates the bowel to empty and prevents constipation but also alters the gut microbiome. Another factor, perhaps the tobacco component diterpenoids, may be responsible for the PD risk-reducing effect.

## Introduction

Parkinson’s disease (PD) is a long-term degenerative disorder of the central nervous system that mainly affects the motor system. The cause of PD is unknown, with both inherited and environmental factors involved. Recently, the gut microbiome has been implicated [1,2].

Tobacco smokers have reduced PD risk. The inverse relationship between smoking and PD is dose-dependent: the more a person smokes, the less chance that he or she will develop PD [3].

Some patients with PD experience constipation long before they develop mobility problems, and constipation is a frequent complaint of people who try to stop smoking. One in six smokers attempting to quit will become constipated; for one in 11, the problem is severe. Constipation is reduced but still reported if nicotine replacement therapy is used [4].

Coffee and tea consumption are associated with reduced PD risk, as well as reduced risk of Alzheimer’s disease (AD) [5,6].

In the present study, we analyzed the relationship between smoking and constipation in subjects with PD and controls. Our aim was to determine whether the effects of smoking and constipation were independent or whether they might be interrelated. To evaluate the relationship, we used a cohort of subjects from the UK Biobank (UKB).

## Materials and methods

Methods

The UK Biobank is a large prospective observational study of men and women. Participants were recruited from across 22 centers located throughout England, Wales, and Scotland between 2006 and 2010 and continue to be longitudinally followed for the capture of subsequent health events [7]. Follow-up health information is provided by linkage to primary care electronic health records, death and cancer registries, and hospital admission records [8]. Some participants had PD at the beginning of the study. Our UK Biobank application was approved as UKB project 57245 (SL and PHR).

Study design and inclusion criteria

Our analysis included all subjects with PD. The diagnosis was ascertained using the 10th revision of the International Classification of Diseases (ICD10 G20). Constipation was recorded in UKB category 100074, medical conditions, data field 20002, non-cancer illness, and self-report code 1599 constipation. Smoking information was from UKB category 100058, data field 1239, and current tobacco smoking. The three valid responses were as follows: 0 - no, 1 - only occasionally, and 2 - yes on most or all days.

Exclusion criteria

We did not include subjects missing any of the inclusion criteria or make corrections for missing data.

Ethics approval

UK Biobank has approval from the Northwest Multi-center Research Ethics Committee (MREC), which covers the UK. It also sought approval in England and Wales from the Patient Information Advisory Group (PIAG) for gaining access to information that would allow it to invite people to participate. PIAG has since been replaced by the National Information Governance Board for Health & Social Care (NIGB). In Scotland, UK Biobank has approval from the Community Health Index Advisory Group (CHIAG).

Data processing was performed using Minerva, a Linux mainframe with Centos 7.6, at the Icahn School of Medicine at Mount Sinai. We used the UK Biobank Data Parser (ukbb parser), a python-based package that allows easy interfacing with the large UK Biobank dataset [9]. SPSS version 25.0 (IBM Corp., Armonk, NY, USA) was used for data analysis (chi-square test, Fisher’s exact test, and logistic regression).

## Results

We analyzed data from 501,174 subjects. The age at enrollment was 56 ± 8 years (mean ± SD). Of the subjects, 56% were women and 44% were men, and 98% were White and British. The subjects were followed for 13.7 ± 2.7 months.

Table 1 shows the smoking history of the 501,174 subjects versus Parkinson’s disease. The decrease in Parkinson’s disease with increased smoking was significant (p < 0.001, two-tailed Fisher’s exact test).

**Table 1:** Smoking history of the 501,174 subjects versus Parkinson’s disease. The three valid responses were no, only occasionally, and yes on most or all days. The decrease in Parkinson’s disease with increased smoking was significant (p < 0.001, two-tailed Fisher’s exact test).

		Parkinson’s disease	
Smoking		No	Yes
No	Count	446,389	1,808
	% within smoking	99.60%	0.40%
Occasionally	Count	13,697	37
	% within smoking	99.70%	0.30%
Yes	Count	39,160	83
	% within smoking	99.80%	0.20%
Total	Count	499,246	1,928
	% within smoking	99.60%	0.40%

Table 2 shows constipation in 501,174 subjects, PD and controls. The increase in constipation in subjects with PD is significant (p = 0.001, two-tailed Fisher’s exact test).

**Table 2:** Constipation in 501,174 subjects, PD and controls. The increase in constipation in subjects with PD is significant (p = 0.001, two-tailed Fisher’s exact test).

		Parkinson’s		Total
Constipation		No	Yes	
No	Count	498,850	1,931	500,781
	% within constipation	99.60%	0.40%	100%
Yes	Count	396	7	403
	% within constipation	98.30%	1.70%	100%
Total	Count	499,246	1,938	501,184
	% within constipation	99.60%	0.40%	100%

Coffee drinking in 501,184 subjects is tabulated in Table 3. PD incidence was lower in subjects who drank caffeinated coffee, but not in subjects who drank decaffeinated coffee or did not drink coffee.

**Table 3:** Coffee drinking in 501,184 subjects. Coffee: none, decaffeinated coffee (any type), instant coffee, ground coffee (includes espresso and filter), and other types of coffee The variability was significant (p < 0.001, two-tailed chi-square).

Coffee		Control	Parkinson’s	Total
None	Count	116,544	467	117,011
	% within column	23.30%	24.10%	23.30%
Decaf	Count	73,965	373	74,338
	% within column	14.80%	19.20%	14.80%
Instant	Count	214,011	792	214,803
	% within column	42.90%	40.90%	42.90%
Ground	Count	87,611	287	87,898
	% within column	17.50%	14.80%	17.50%
Other	Count	7,115	19	7,134
	% within column	1.40%	1%	1.40%
Total	Count	499,246	1,938	501,184
	% within column	100%	100%	100%

Table 4 displays logistic regression with 95% confidence intervals (CIs). Sex, age, constipation, smoking, drinking coffee, and drinking tea were the independent variables, and PD present or absent was the dependent variable. The PD odds ratio (OR) for males was 1.790 times that for females, indicating that PD is more common in men. The risk of PD increased by 1.140 with every year of age. Constipation increased the risk of PD by 4.043. Smoking (yes or no) diminished PD risk by 0.772. Drinking coffee was associated with a reduced risk of PD (OR: 0.815). Drinking tea reduced PD risk by 0.979 for each cup per day. The effects of sex, age, constipation, smoking, drinking coffee, and drinking tea were independent and significant.

**Table 4:** Logistic regression with 95% confidence intervals (lower bound (LB) and upper bound (UB)).

	95% LB	OR	95% UB	p value
Sex	1.629	1.790	1.966	<0.001
Age	1.131	1.140	1.149	<0.001
Constipation	1.901	4.043	8.599	<0.001
Smoking	0.690	0.772	0.863	<0.001
Drinking coffee	0.730	0.815	0.909	<0.001
Drinking tea	0.962	0.979	0.997	0.017

## Discussion

The gut microbiome’s influence on the brain is being actively studied. In one report, mice without gut bacteria were less anxious than their healthy equivalents. In 2017, a systematic review and meta-analysis demonstrated that people with constipation are at higher risk of developing PD compared with those without and that constipation can predate PD diagnosis by over a decade [10]. The relatively low incidence of constipation we found in UKB patients with PD (1.7%) could be attributed to the healthy lifestyle of the UKB subjects compared with the general British population [11]. The present study did not investigate the influence of the gut microbiome on PD and smoking.

In PD, the protein α-synuclein misfolds. The misfolded protein causes more misfolds until harmful clumps of Lewy bodies form in the brain. Gut bacteria can produce proteins that have a similar structure to α-synuclein, so bacterial proteins might be providing a template for misfolding [12]. A strain of *Escherichia coli* in the gut makes a protein, curli, which can stimulate other proteins, including α-synuclein, to misfold. The misfolded proteins might transmit the aberration up the vagus nerve to the brain, where misfolded α-synuclein is linked to PD symptoms [13].

Stomach ulcers were once treated by removing all or part of the vagus nerve to reduce acid production in the stomach. Interestingly, people who had undergone this procedure had less risk of Parkinson’s disease [14].

The reasons for the inverse association between tobacco smoking and PD are not fully understood. Some studies have suggested that nicotine, such as caffeine, may have neuroprotective properties and stimulate the release of dopamine. However, high-dose transdermal nicotine in patients with PD was not beneficial [15]. Therefore, other components of tobacco may be responsible for the neuroprotective effect. Although smoking may reduce the risks of PD, the reduction in risk is small, and most likely, the harmful effects of tobacco outweigh the protective effects.

Tobacco has a long history of medicinal applications [16]. The leaves and juice were much used for skin disorders, possibly including basal cell cancer. Of most interest in Parkinson’s disease are the diterpenes in tobacco.

Diterpenes are a class of chemical compounds composed of four isoprene units. They are biosynthesized by plants, animals, and fungi via the 3-hydroxy-3-methylglutaryl coenzyme A (HMG-CoA) reductase pathway, with geranylgeranyl pyrophosphate being a primary intermediate. Diterpenes form the basis for biologically important compounds such as retinol, retinal, and phytol. They are antimicrobial, neuroprotective, and anti-inflammatory [17,18]. Diterpenes are present in coffee and tea, as well as in tobacco leaves, and coffee and tea drinkers have reduced PD risk, as well as AD risk [6,19].

Tobacco plants use nicotine and diterpenoids to protect themselves from pests such as tobacco hornworms. Diterpenoids are extremely poisonous. Tobacco plants have evolved a complex process to maintain a balance between self-defense and self-poisoning. Two cytochrome P450 enzymes act in the biosynthesis pathway of 17-hydroxygeranyllinalool diterpene glycosides in wild tobacco (*Nicotiana attenuata*) to prevent the accumulation of hazardous diterpene derivatives. After ingestion, the same diterpene derivatives develop in an insect herbivore, causing toxicity by blocking sphingolipid production in both the plant and the insect [20]. Diterpenes are active against Parkinson’s disease and other forms of neurodegeneration and have been suggested as therapeutic agents [21].

Our results agree with previous studies that drinking coffee, but not decaffeinated coffee, and tea protect against PD [22]. The protection may be partly due to caffeine, which is both a central nervous system stimulant and neuroprotectant [5,23-25]. However, the diterpene content of coffee and tea may contribute to the PD protective effect, as well as a protective effect against AD [26], since decaffeinated coffee has markedly reduced levels of diterpene esters [27].

Limitations

Our study is limited due to the fact that it is retrospective and we were unable to assess the severity or duration of PD relative to constipation and cigarette smoking. We had only ICD10 G20 and no other details of the PD.

## Conclusions

Our analysis suggests that the favorable effect of smoking on PD is independent of the detrimental effect of constipation. Smoking reduces PD risk because it not only stimulates the bowel to empty and prevents constipation but also alters the gut microbiome. Another factor, perhaps the tobacco component diterpenoids, may be responsible for the PD risk-reducing effect.

The search for PD therapy in tobacco should not begin and end with nicotine patches. Further studies are certainly warranted.
